# Variation Patterns of Hemoglobin Levels by Gestational Age during Pregnancy: A Cross-Sectional Analysis of a Multi-Center Retrospective Cohort Study in China

**DOI:** 10.3390/nu15061383

**Published:** 2023-03-13

**Authors:** Mengxing Sun, Tingfei Gu, Tianchen Wu, Xiaoli Gong, Xiaona Li, Jiaqi Huang, You Li, Yangyu Zhao, Huifeng Shi, Yuan Wei

**Affiliations:** 1 Department of Obstetrics and Gynecology, Peking University Third Hospital, Beijing 100191, China; 2 National Clinical Research Centre for Obstetrics and Gynecology, Beijing 100191, China; 3 Health Science Center, Peking University, Beijing 100083, China; 4 National Centre for Healthcare Quality Management in Obstetrics, Beijing 100191, China; 5 Department of Pharmacy, Peking University Third Hospital, Beijing 100191, China

**Keywords:** anemia, hemoglobin, pregnancy, reference, gestational age, iron deficiency

## Abstract

Background: Pregnancy anemia is a global health concern. However, to our knowledge, there still has little consensus on the reference value of hemoglobin levels. Particularly, little evidence from China was accessible in most existing guidelines. Objective: To evaluate hemoglobin levels and anemia prevalence of pregnant women in China and offer evidence for anemia and its reference values in China. Methods: A multi-center retrospective cohort study was conducted among 143,307 singleton pregnant women aged 15–49 at 139 hospitals in China, with hemoglobin concentrations routinely tested at each prenatal visit. Subsequently, a restricted cubic spline was performed to reveal a non-linear variation of hemoglobin concentrations during the gestational week. The Loess model was used to describe the changes in the prevalence of different degrees of anemia with gestational age. Multivariate linear regression model and Logistic regression model were applied to explore influencing factors of gestational changes in hemoglobin level and anemia prevalence, respectively. Results: Hemoglobin varied nonlinearly with gestational age, and the mean hemoglobin levels decreased from 125.75 g/L in the first trimester to 118.71 g/L in the third trimester. By analyzing hemoglobin levels with gestational age and pregnancy period, we proposed new criteria according to 5th percentile hemoglobin concentration in each trimester as a reference for anemia, with 108 g/L, 103 g/L, and 99 g/L, respectively. According to WHO’s criteria, the prevalence of anemia sustainably increased with gestational age, with 6.2% (4083/65,691) in the first trimester, 11.5% (7974/69,184) in the second trimester and 21.9% (12,295/56,042) in the third trimester, respectively. In subsequent analysis, pregnant women in non-urban residents, multiparity, and pre-pregnancy underweight tended to have lower hemoglobin levels. Conclusions: This research, the first large-sample study to present a set of gestational age-specific reference centiles for hemoglobin levels in China, could be used to obtain a better understanding of the overall levels of hemoglobin in Chinese healthy pregnant women and ultimately offer clues for a more precise hemoglobin reference value of anemia in China.

## 1. Introduction

Anemia is a state in which the number of red blood cells or hemoglobin is not reduced enough to meet the physiological needs of the body [1]. Particularly, gestational anemia, associated with increased risk of cesarean section, maternal mortality, reduced birthweight, and preterm birth [1,2,3,4], remains a global health concern [5,6].

Appropriate guidelines for the measurement of hemoglobin and the definition of anemia are crucial for both clinical and public health medicine. The most widely used guideline of anemia is WHO’s recommendations, with severe, moderate, and mild anemia for pregnant women referring to hemoglobin concentrations of less than 70 g/L, 70 to 99, and 100 to 109 g/L, respectively. It should be emphasized that WHO’s guideline was first proposed based on five studies of predominantly white populations in Europe and North America, with little available data from China. However, reference for anemia should particularly consider complexities across different populations, especially with racial and environmental factors [1]. Moreover, although other guidelines have already recommended a hemoglobin cutoff lower than 110 g/L to define anemia during pregnancy, there is a lack of evidence in China, and therefore, further work is needed to validate them.

In fact, several studies have already reported similar or even lower risks of low birthweight and stillbirth in pregnant females with mild anemia compared with those who had normal hemoglobin concentrations according to WHO recommendations [7,8]. Particularly, a national study in China reported that mild anemia in Chinese pregnant women is associated with improved maternal and fetal survival and fetal growth [9]. Conversely, studies have already reported a U-shaped curve for risk associated with maternal hemoglobin, iron status, or iron supplementation, with routine iron supplements bringing higher risks of SGA (Small for Gestational Age) and hypertension disorder [8,10]. Thus, although anemia remains one of the most common laboratory diagnoses, consensus on the hemoglobin threshold below which it should be defined is limited, especially when it comes to China [9].

In response to these issues, this study, by depicting the variation curve of hemoglobin levels with gestational age, aimed to provide clues for a more precise reference range of maternal hemoglobin levels during pregnancy in China.

## 2. Methods

### 2.1. Study Design and Data Collection

We used the data from a multi-center prospective cohort study designed to explore the correlation between serum vitamins A and E during pregnancy and preeclampsia from 2015 to 2020, recruiting pregnant women who received routine prenatal care during whole pregnancy at 180 hospitals in 23 provinces across three geographical regions in China. In order to guarantee data quality, at least one tertiary maternity hospital was invited as the research center in each province. This study collected results of maternal blood routine examinations and correspondent medical records in at least one follow-up during the 1st, 2nd, and 3rd trimesters of pregnancy.

We conducted a multi-center retrospective study and a secondary analysis based on the prospective cohort. A total of 143,307 singleton pregnant women, aged 15–49 were recruited in this study. We used data whose records contained at least one hemoglobin measurement during 5–41 gestational weeks. Cases of pregnant women who did not have credible results of hemoglobin concentration (<30 g/L or >180 g/L), were conceived by assisted reproductive technology, or had pregnancy complications (hypertension, preeclampsia, diabetes, gestational diabetes, intrahepatic cholestasis, abnormal amniotic fluid, premature rupture of membranes, threatened abortion, etc.) were excluded from this study.

All the procedures of this study were reviewed and approved by the Peking University Third Hospital Medical Science Research Ethics Committee (IRB00006761-2015277).

### 2.2. Traditional RANGE of Hemoglobin Measurement and Maternal Anthropometrics

Hemoglobin concentrations were routinely tested at the local laboratory at each prenatal visit. Anemia for pregnant women was classified by hemoglobin levels according to WHO definitions: severe anemia (<70 g/L), moderate anemia (70~100 g/L), and mild anemia (100~110 g/L).

Provinces were divided into three geographical regions: eastern China (Beijing, Tianjin, Hebei, Liaoning, Shandong, Jiangsu, Zhejiang, and Guangdong), central China (Anhui, Henan, Shanxi, Hubei, Hunan, Jilin, and Heilongjiang), and western China (Chongqing, Yunnan, Sichuan, Guangxi, Qinghai, Ningxia, Shaanxi, and Inner Mongolia). Age, ethnic origin, education, Hukou (urban residents, rural residents, or rural-to-urban migrants), primigravida, labor year, height, and weight prior to pregnancy were extracted from medical records. Underweight, normal BMI, overweight, and obesity were defined as a BMI of <18.5,18.5–23.9, 24–27.9, and ≥28, respectively, by using the diagnostic criteria in Chinese adults.

### 2.3. Statistical Analysis

We summarized the baseline characteristics of pregnant women (Table 1). We calculated the mean and SD of hemoglobin concentration and anemia prevalence (refer to WHO criteria) of each gestational week. Pearson’s chi-square test was applied to categorical variables and the one-way ANOVA test was applied to continuous variables for comparison. Restricted cubic spline was performed to reveal a non-linear variation of hemoglobin concentrations during the gestational week. Loess-model was used to describe the changes in the prevalence of different degrees of anemia with gestational age. Multivariate linear regression model and logistic regression were applied to explore influencing factors of gestational changes in hemoglobin level and anemia prevalence in different trimesters, respectively. All statistical analyses were performed with SPSS software version 26.0, SAS software, version 9.0, and the R statistical software, version 3.6.2. *p* < 0.05 was regarded as statistically significant.

## 3. Results

**Figure 1:**
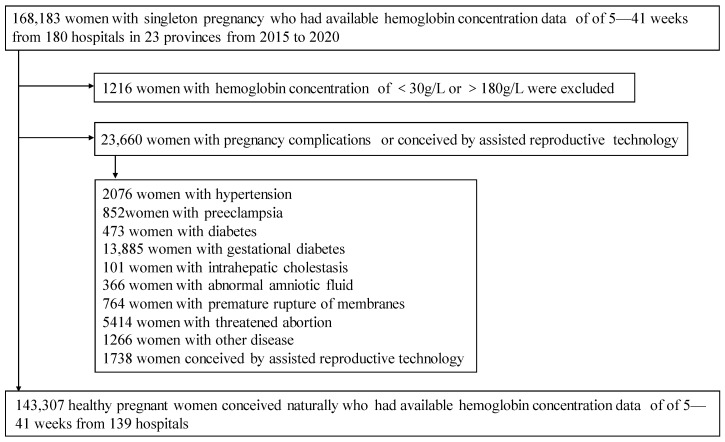
Study profile.

A total of 143,307 pregnant women with required data from 139 hospitals were included (Figure 1).

### 3.1. Baseline Characteristics

In our study, the major labor years of participants were between 2015 and 2018(93.4%). The mean age of pregnant women was 28.78 (±4.48) years old. Most participants lived in urban areas (69.6%) and attained an educational of level more than high school (62.7%). The majority of participants were divided into the normal pre-pregnancy BMI group (69.7%) and were from eastern China (54%). The proportion of primigravid and multipara was 55.4% and 44.6%, respectively; 97.8% of participants’ ethnicity were Han and 21.8% of participants’ ethnicity were minorities (Table 1).

#### 3.1.1. Hemoglobin Levels during Pregnancy

**Figure 2:**
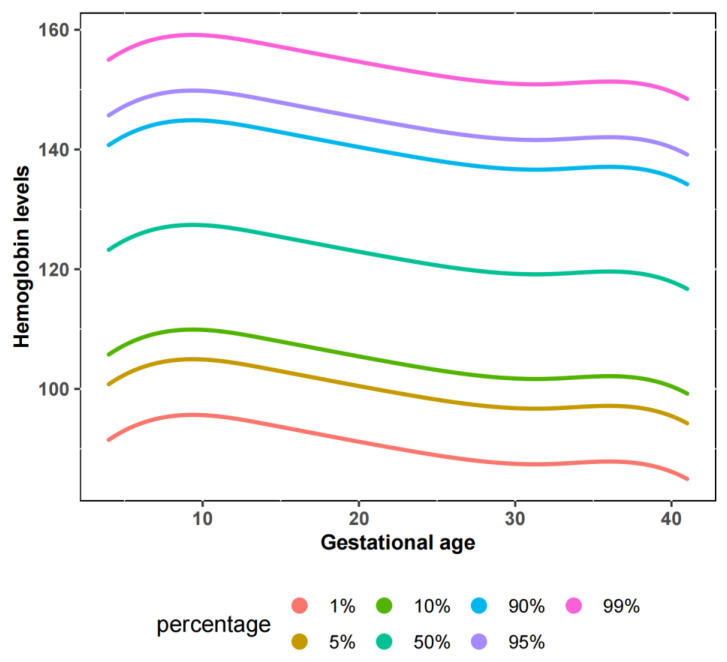
Estimated 1st, 5th, 10th,50th, 90th, 95th, and 99th percentiles for hemoglobin concentration by gestational age. Restricted cubic spline was performed to reveal a non-linear variation of hemoglobin concentrations during the gestational week.

Results of hemoglobin concentration during 5–41 weeks of 143,307 pregnant women were calculated, in which 65,691, 69,184, and 56,042 participants had effective hemoglobin values in the first, second, and third trimesters, respectively. The mean (SD) and percentile of hemoglobin concentrations and the anemia prevalence according to gestational age were displayed in Table 2. The median hemoglobin concentration was highest (128 [IQR 121–135] g/L) in the 8th gestational week and 119 (IQR 110–116) g/L in the 40th gestational week. We modeled gestational age by applying restricted cubic splines to allow the pattern of hemoglobin concentration to vary in a smooth manner across the whole period. The estimated 1st, 5th, 10th, 50th, 90th, 95th, and 99th percentiles for hemoglobin concentration by gestational age were displayed in Figure 2. To assess the validity of the model, we visually compared the predicted mean and SD with the crude data and calculated the percentage of hemoglobin measurements that fell within the predicted limits for 1 and 2 SDs (where 77.6% and 96.1%, respectively, would be expected in a perfect model). We supposed that the 5th percentile hemoglobin concentration level is a new definition of maternal anemia in China for avoiding overtreatment.

#### 3.1.2. Prevalence of Anemia among Pregnant Women

**Figure 3:**
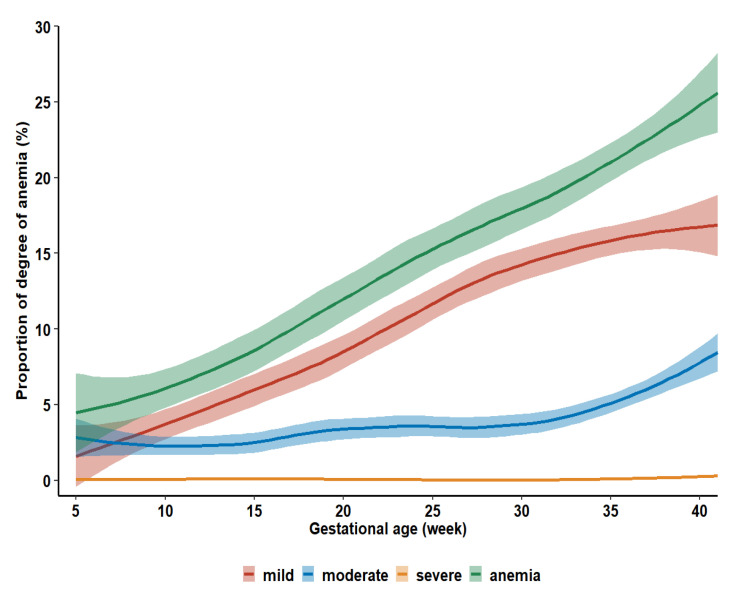
The estimated proportion of degree of anemia by gestational age. Restricted cubic spline was performed to reveal a non-linear variation of anemia during the gestational week.

The prevalence of anemia of all degrees was presented in a slightly increasing trend by gestational age shown in Figure 3.

**Figure 4:**
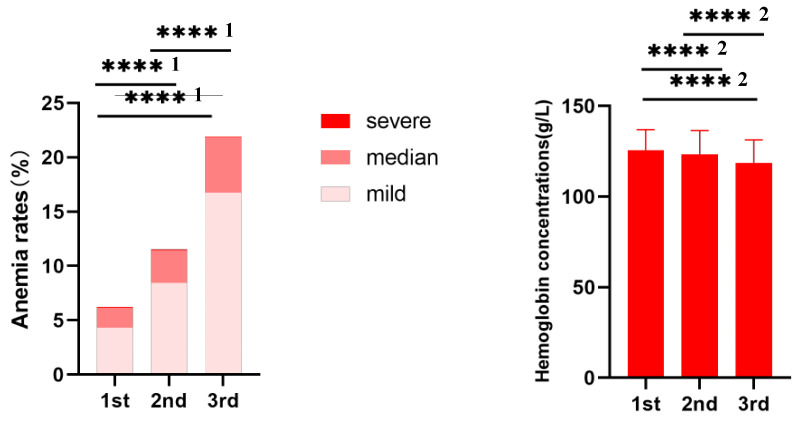
Hemoglobin concentration and the prevalence of anemia in pregnant women with respect to trimester. ****^1^: *p <* 0.001, from One-way ANOVA. ****^2^: *p <* 0.001, from Pearson’s chi-square test.

We calculated hemoglobin concentrations and the prevalence of anemia according to trimesters in WHO criteria and our definition. As shown in Table 3 and Figure 4, hemoglobin tended to decrease with advancing trimesters from 125.75 g/L in the first trimester to 118.71 g/L in the third trimester. The prevalence of anemia was 6.2% (4083/65,691) in the first trimester, 11.5% (7974/69,184) in the second trimester, and 21.9% (12,295/56,042) in the third trimester (Table 4) by WHO criteria. Mild anemia was predominant in all trimesters of pregnancy. However, the anemia prevalence was 4.8% in the first trimester, 4.9% in the second trimester, and 4.4% in the third trimester by regarding hemoglobin level below the 5th percentile hemoglobin concentration reference in each trimester as anemia.

### 3.2. Subgroup Percentages of Maternal Hemoglobin Concentration and Anemia

Table 4 and Table 5 showed hemoglobin concentrations and the prevalence of anemia in each trimester of subgroups classified by different characteristics, including year, area, Hukou, age group, education, and *p*-BMI group.

### 3.3. Subgroup Logistic Regression Analyses of Hemoglobin Levels and Anemia

As shown in Table 6 the multivariate linear regression analysis identified the associated factors with hemoglobin concentrations in each trimester. The association between labor year and hemoglobin concentration varied by gestational age. The labor year was positively associated with hemoglobin concentration in the first trimester, while negatively associated with hemoglobin concentration in the second and third trimesters. Pregnant women in eastern China had higher serum hemoglobin concentrations during pregnancy than those in central China and western China in the first trimester (B, −2.146 [95%CI, −2.392, −1.899]), (B, −2.664 [95%CI, −2.926, −2.402]), but this trend gradually disappeared in the third trimester. Compared with urban pregnant women, migrants, and rural pregnant women had lower hemoglobin concentrations during pregnancy. Older pregnant women tended to have higher hemoglobin concentration, with *p* < 0.001, 0.078(0.056, 0.101) in the second trimester and *p* < 0.001, 0.115(0.090, 0.140) in the third trimester. The hemoglobin level of multiparous women was significantly lower than that of primiparous women. Compared with normal pre-pregnancy BMI, the hemoglobin level of underweight pregnant women was significantly lower, and the hemoglobin level of obese and overweight pregnant women was significantly higher.

Table 7 presented the associated factors with the anemia prevalence in each trimester. Pregnant women in western China had a higher risk of anemia (OR, 1.788 [95%CI, 1.652–1.935]) than those in eastern China in the first trimester, but this trend reverse in the second (OR, 0.811 [95%CI, 0.763–0.863]) and the third trimester (OR, 0.938 [95%CI, 0.895–0.983]). Pregnant women residing in central China suffered higher risk than those in eastern China. Older pregnant women had a higher anemia prevalence in the first trimester (OR, 1.027 [95%CI, 1.109–1.035]), while had a lower risk of anemia in the second (OR, 0.994 [95%CI, 0.988–1.000]) and the third trimester (OR, 0.987 [95%CI, 0.982–0.992]). The following factors were identified independently associated with a higher risk of anemia during the whole pregnancy: multiparity, pre-pregnancy underweight, and higher education level.

## 4. Discussion

### 4.1. Summary

This study innovatively demonstrated variations in hemoglobin levels and anemia prevalence by gestational age in 143,307 singleton pregnant women. Hemoglobin varied nonlinearly with gestational age, and the mean hemoglobin levels decreased from 125.75 g/L in the first trimester to 118.71 g/L in the third trimester. By analyzing hemoglobin levels with gestational age and pregnancy period, we proposed new criteria according to 5th percentile hemoglobin concentration in each trimester as a reference for anemia, with 108 g/L, 103 g/L, and 99 g/L, respectively. According to WHO’s criteria, the prevalence of anemia sustainably increased with gestational age, with 6.2% (4083/65,691) in the first trimester, 11.5% (7974/69,184) in the second trimester and 21.9% (12,295/56,042) in the third trimester respectively while anemia prevalence was 4.8% in the first trimester, 4.9% in the second trimester and 4.4% in the third trimester according to our new reference. We also depicted hemoglobin concentration levels in different trimesters and explored their associated factors, with non-urban residents, multiparity, and pre-pregnancy underweight associated with lower hemoglobin levels.

### 4.2. Comparisons and Applications

In our study, we mainly found that hemoglobin levels demonstrated natural fluctuations in hemoglobin levels by trimester, due to fetal and maternal physiological demands. Generally, it is recognized that there is a normal 1.0 g/dL decrease in hemoglobin in 1st and 3rd trimesters, with hemoglobin concentrations diminishing an additional 0.5 g/dL in 2nd trimester of pregnancy [11].

In subgroup logistic analysis, we found that pregnant women in eastern China had higher serum hemoglobin concentrations during pregnancy than those in central China and western China in the first trimester. Considering that eastern China is more developed than other areas of China, differences in the 1st trimester of pregnancy which reflects the basic iron amount probably arise from different levels of local economic development, lifestyle, and diet [12].

As a reference to maternal anemia, according to WHO’s criteria, the prevalence of anemia was 6.2%, 11.5%, and 21.9% in 1st, 2nd, and 3rd trimesters of pregnancy, respectively, this was similar to the prevalence of anemia in China reported earlier [9,12,13]. It is remarkable that mild anemia by WHO’s criteria refers to 100 to 109 g/L hemoglobin levels during pregnancy. However, in our new recommendation, the range of mild anemia by WHO mainly fell into a reference for non-anemia, similar to studies that proposed hemoglobin cutoff lower than 110 g/L to define anemia during pregnancy in other countries. In fact, our previous nationwide study [9] has already reported that mild anemia by WHO’s recommendations is associated with decreased risks of fetal growth restriction and stillbirth in Chinese pregnant women. This finding was similar to several previous studies [4,14,15,16,17,18,19]. Increasing evidence shows that the downregulation of hepcidin and upregulation of erythropoietin related to iron deficiency may have protective effects on the cardiovascular system and other organs [20,21]. A relatively low level of hemoglobin during pregnancy probably reflects benign plasma volume expansion, which in turn reduces blood viscosity, increases uteroplacental blood flow and uteroplacental perfusion, accompanied by cardiovascular changes including increased cardiac output and decreased peripheral resistance [22], thus benefiting maternal survival and facilitate fetal growth and development. Furthermore, previous studies have already reported that iron deficiency anemia was found to be associated with increased placental size and angiogenesis as well as upregulation of placental transfer systems, a physiological change serving to facilitate fetal growth and survival, in favor of fetal oxygen and nutrient supplies and ultimately [23,24].

In all, considering that mild anemia by WHO has protective effects on fetal outcomes, our study indicated that, for Chinese pregnant women, the existing WHO recommendations might need some supplementation.

Moreover, pregnant women with normal hemoglobin levels were vulnerable to adverse effects of excessive interferences, as WHO recommends daily routine supplementation with 30–60 mg elemental Fe/d (plus 400 μg folic acid) throughout pregnancy. In fact, although most studies reported that supplementation of anemic women with iron reduces the rate of anemia at term [25], the adverse effect of overloaded iron and hemoglobin has already been reported [26], with higher risks of preeclampsia, prematurity, gestational diabetes (GDM) and fetal growth restriction among iron-replete pregnant women [10,27,28,29,30,31]. Astonishingly, we found that our new reference for pregnant anemia will reduce anemia, particularly excessive interference of more than 1.4%, 6.7%, and 17.5% of pregnant women in China in the 1st, 2nd, and 3rd trimesters of pregnancy, respectively.

Furthermore, although there did exist studies about maternal anemia in China, those studies only reported anemia prevalence, with little mention of precise hemoglobin levels. Therefore, this is the first to offer a pure perspective on anemia and hemoglobin levels of Chinese pregnant women, and its proposed progress might be further applied to or validate studies in other countries.

It should be stressed that our study almost did not include moderate and severe anemia (Figure 2), so the new model has little influence on them. In fact, higher levels of anemia are associated with negative perinatal outcomes, including postpartum hemorrhage and hypovolemia [32]. We believe that interventions for moderate to severe anemia should be recommended and that monitoring and prevention of potential adverse outcomes are needed for pregnant females with anemia. Meantime, the lower threshold of anemia offers an opportunity for reducing unnecessary treatment and patients’ anxiety, better guarding the progress of a pregnancy.

### 4.3. Limitations

It should be noted that due to certain reasons, the database of this study lacked accurate information on perinatal outcomes. Therefore, we could not evaluate relationships between hemoglobin levels and perinatal outcomes. However, our previous study might supplement this gap to some extent, offering a view of the relationships between anemia rates and perinatal outcomes [9]. Furthermore, from a statistical perspective, it seems that this study design is enough to offer a reliable threshold for hemoglobin levels based on five percent of all pregnant women. In a subsequent study, we will further this research and verify this result.

Despite controlling for many covariates in the multivariable-adjusted analyses, we did not measure several important factors, especially iron supplementation and transfusions of blood products during pregnancy, which may have confounded the observed associations because of the retrospective nature of this study. Furthermore, hemoglobin levels were also influenced by dietary habits. The usage of iron supplements/drug information and dietary habits were not included in most previous research. We are aware of this and will fill this gap in subsequent single-center studies.

In addition, we did not take specific residence into consideration information in data collection, therefore it would not be possible to adjust for hemoglobin levels in pregnant women at high altitudes. Most subjects lived in provinces with average altitudes lower than 1000 m, where there is no need to adjust hemoglobin levels according to WHO guidelines. In the following multi-center study, we will collect the specific residence of the subjects and adjust the hemoglobin level according to the altitude of the residence before analysis.

Moreover, we could not ensure appropriate standardization of hemoglobin measurements across different hospitals and regions, which might bring bias to the results, but it should be stressed that there already has a growing tendency that clinical laboratory results will achieve inter-accreditation among different hospitals in China.

## 5. Conclusions

This is the first to offer a pure perspective on anemia and hemoglobin levels of Chinese pregnant women, and its proposed progress might be further applied to or validate studies in other countries. In this study, we proposed new criteria according to 5th percentile hemoglobin concentration in each trimester as a reference for anemia, with 108 g/L, 103 g/L, and 99 g/L, respectively. According to this reference, we found a potential reduction of anemia excessive interference of more than 1.4%, 6.7%, and 17.5% of pregnant women in China in the 1st, 2nd, and 3rd trimesters of pregnancy, respectively.

Considering that there was little evidence according to maternal anemia from China, more cross-sectional studies and precise guidelines based on them should be considered essential in the effort to monitor maternal hemoglobin status better.

## Figures and Tables

**Table 1:** Basic characteristics of pregnant women.

Characteristics	*n* (%)
Year	
2015	22,824 (15.9)
2016	35,706 (24.9)
2017	42,555 (29.7)
2018	32,773 (22.9)
2019	8414 (5.9)
2020	1035 (0.7)
Area	
Eastern China	77,441 (54.0)
Central China	26,523 (18.5)
Western China	39,343 (27.5)
Hukou	
Urban residents	99,767 (69.6)
Migrants	22,856 (15.9)
Rural residents	20,684 (14.4)
Age, mean (SD)	28.78 (4.48)
Age group	
15–19	1347 (1.0)
20–24	20,676 (14.4)
25–29	66,386 (46.3)
30–34	38,506 (26.9)
35–39	13,981 (9.8)
40–49	2411 (1.7)
Ethnics	
Han	140,088 (97.8)
Others ^a^	3219 (2.2)
Education	
High school	35,938 (25.1)
College	46,994 (32.8)
Master	6943 (4.8)
Others	53,432 (37.3)
Gravidity	
Primigravid	79,412 (55.4)
Multipara	63,895 (44.6)
Pre-pregnancy BMI ^b^, mean (SD), kg/m^2^	21.44 (3.31)
Pre-pregnancy BMI group	
Normal weight(18.5 ≤ BMI < 24)	99,886 (69.7)
Underweight(BMI < 18.5)	19,825 (13.8)
Overweight (24 ≤ BMI < 28)	17,679 (12.3)
Obesity(BMI ≥ 28)	4698 (3.3)
Unknown	1219 (0.9)

^a^ Because there are more than 50 ethnic groups in China, other groups are not listed individually; ^b^ BMI Body Mass Index.

**Table 2:** Hemoglobin concentrations (g/L) and the prevalence of anemia (%) according to gestational age.

Week	*n*	Hemoglobin Concentrations(g/L)														Anemia(%)
		Mean	SD	Median	1	3	5	10	25	50	75	90	95	97	99	
1st trimester	65,691	125.75	11.17	126	94	103	108	112	120	126	133	140	143	146	150	6.2
5	1515	121.59	8.58	120	87	101	110	120	120	120	120	130	135	143	149	1.9
6	979	124.52	12.76	124	86	89	101	111	120	124	133	140	144	146	149	6.6
7	2302	125.90	12.44	126	85	95	106	112	120	126	134	141	145	147	154	7.0
8	7277	127.84	11.17	128	95	105	110	115	121	128	135	141	145	148	152	4.4
9	5626	127.20	11.67	128	92	99	108	113	120	128	135	141	145	147	151	5.9
10	6538	126.18	11.68	127	92	96	107	112	120	127	134	140	144	147	151	6.6
11	8127	125.64	11.43	126	94	98	107	112	119	126	133	139	143	147	152	6.8
12	25,653	125.49	10.56	126	97	102	108	112	119	126	132	138	142	145	149	6.1
13	7711	124.24	11.36	124	93	99	106	111	118	124	131	139	143	146	149	8.2
2nd trimester	69,184	123.47	13.01	123	90	99	103	108	115	123	132	141	146	148	154	11.5
14	3904	123.96	11.90	124	90	98	105	110	117	124	131	139	143	146	150	8.6
15	5782	126.24	12.79	126	88	100	106	112	118	126	134	142	147	149	154	6.8
16	8976	126.62	12.13	127	93	103	107	112	119	127	134	142	146	148	154	6.5
17	6758	125.61	12.64	125	92	101	105	111	118	125	134	143	147	148	153	8.3
18	5125	125.79	13.82	126	88	99	103	110	117	126	135	144	147	148	154	9.3
19	2728	125.11	14.91	124	87	95	101	108	116	124	135	145	149	152	157	11.7
20	2786	122.93	14.19	122	85	94	101	106	114	122	132	142	147	149	155	14.2
21	3846	121.56	13.74	120	88	94	100	106	113	120	130	141	146	148	154	16.5
22	3190	121.98	13.93	121	86	93	100	106	113	121	131	141	146	148	152	15.2
23	3317	122.99	14.27	123	86	94	99	105	114	123	132	142	147	149	155	14.5
24	9481	122.04	11.79	122	93	96	103	108	115	122	130	137	142	146	150	12.1
25	5808	121.73	12.27	121	91	94	103	108	114	121	130	137	143	147	153	13.5
26	5721	120.93	11.97	120	90	97	102	107	113	120	128	136	142	146	151	14.7
27	4363	121.17	12.14	120	92	98	103	107	113	120	128	136	142	147	152	14.4
3rd trimester	56,042	118.71	12.64	118	89	96	99	104	110	118	126	134	141	145	154	21.9
28	12,159	117.91	10.78	117	95	99	102	105	111	117	124	131	136	141	147	21.0
29	2621	122.12	12.81	122	94	99	102	106	113	122	130	140	145	147	156	15.4
30	3746	121.27	12.29	121	93	97	101	106	113	121	129	136	142	145	153	16.2
31	3301	119.36	12.56	120	90	95	100	104	111	120	127	134	141	147	155	21.1
32	7713	117.97	11.67	118	90	95	99	103	110	118	126	132	136	139	149	22.4
33	3728	118.62	12.66	118	90	95	99	103	110	118	126	134	140	146	156	22.7
34	6140	120.30	12.28	120	92	98	101	106	112	120	128	136	142	146	149	17.9
35	3207	120.47	12.85	120	88	95	100	105	112	120	128	137	142	146	153	17.7
36	7191	118.02	11.62	118	92	97	100	104	110	118	125	132	137	142	149	22.0
37	3656	119.39	12.93	119	87	94	100	104	111	119	127	135	142	146	154	20.6
38	2677	119.10	13.94	119	83	89	98	102	111	119	127	136	143	148	157	21.6
39	2966	117.30	14.41	118	80	86	93	99	109	118	126	135	141	146	155	25.8
40	2981	117.86	13.60	119	84	89	96	101	110	119	126	133	141	146	154	24.5
41	542	121.75	18.57	119	79	90	95	100	109	119	134	151	157	158	160	26.9

**Table 3:** Hemoglobin concentrations (g/L) and the prevalence of anemia (%) according to trimesters.

Gestational Period	*n*	Hb, X(SD), g/L	Prevalence of Anemia, % (WHO Criteria)			
			Total	Mild	Moderate	Severe
1st trimester	65,691	125.75 (11.17)	6.2	4.3	1.9	0.1
2nd trimester	69,184	123.47 (13.01)	11.5	8.4	3.0	0.1
3rd trimester	56,042	118.71 (12.63)	21.9	16.7	5.1	0.1

**Table 4:** The hemoglobin concentration in subgroups according to trimester.

Characteristics	*n*	Hemoglobin Concentrations ^a^		
		First Trimester	Second Trimester	Third Trimester
Year				
2015	22,824	127.95(127.78,128.11)	122.27(122.06,122.49)	117.67(117.47,117.87)
2016	35,706	125.40(125.25,125.55)	123.06(122.88,123.24)	117.03(116.87,117.20)
2017	42,555	125.09(124.94,125.24)	121.52(121.31,121.72)	118.14(117.93,118.36)
2018	32,773	125.06(124.81,125.31)	124.53(124.33,124.72)	121.11(120.82,121.40)
2019	8414	121.87(120.75,122.98)	127.50(127.13,127.87)	124.97(124.44,125.50)
2020	1035	114.12(105.80,122.45)	130.95(130.37,131.53)	130.39(129.66,131.12)
Area				
East	77,441	127.27(127.14,127.41)	124.01(123.89,124.14)	118.89(118.75,119.03)
Central	26,523	124.81(124.68,124.95)	120.03(119.80,120.27)	118.28(117.92,118.65)
West	39,343	124.17(123.99,124.34)	123.98(123.79,124.18)	118.54(118.36,118.72)
Hukou				
Urban residents	99,767	125.78(125.68,125.87)	122.98(122.86,123.09)	119.05(118.93,119.17)
Migrants	22,856	126.30(126.01,126.59)	126.64(126.42,126.87)	118.75(118.39,119.10)
Rural residents	20,684	125.15(124.91,125.39)	120.92(120.67,121.17)	117.14(116.88,117.40)
Age group				
15–19	1347	124.70(123.64,125.77)	124.21(123.18,125.25)	117.40(116.21,118.60)
20–24	20,676	125.16(124.94,125.39)	122.73(122.47,122.98)	118.08(117.79,118.38)
25–29	66,386	126.11(125.98,126.23)	123.42(123.28,123.56)	118.82(118.66,118.97)
30–34	38,506	125.60(125.43,125.76)	123.79(123.60,123.98)	118.68(118.48,118.87)
35–39	13,981	125.48(125.19,125.77)	123.83(123.51,124.15)	119.17(118.82.119.51)
40–49	2411	124.25(123.44,125.06)	124.20(123.40,125.00)	119.74(118.82,120.67)
Ethnics				
Han	140,088	125.74(125.65,125.83)	123.45(123.35,123.54)	118.72(118.62,118.83)
Others ^b^	3219	125.98(125.39,126.58)	124.76(124.11,125.42)	118.36(117.70,119.03)
Education				
High school	35,938	125.30(125.13,125.47)	122.60(122.39,122.81)	117.91(117.69,118.14)
College	46,994	126.27(126.12,126.42)	123.55(123.37,123.72)	119.78(119.60,119.96)
Master	6943	126.28(125.97,126.59)	124.69(124.29,125.10)	117.23(116.89,117.58)
Other	53,432	125.48(125.34,125.63)	123.77(123.62,123.92)	118.42(118.25,118.59)
Primigravida				
Yes	79,412	126.02(125.90,126.15)	124.75(124.62,124.88)	120.14(119.98,120.29)
No	63,895	125.45(125.33,125.57)	121.67(121.53,121.81)	117.21(117.07,117.34)
p-BMI group				
18.5~23.99	99,886	125.46(125.36,125.56)	123.58(123.46,123.69)	118.85(118.72,118.98)
<18.5	19,825	124.30(124.08,124.53)	123.13(122.87,123.39)	117.90(117.61,118.18)
24~27.99	17,679	128.05(127.81,128.28)	123.68(123.41,123.95)	119.01(118.73,119.29)
>=28	4698	129.74(129.26,130.21)	123.12(122.66,123.57)	118.43(117.91,118.95)
Unknown	1219	123.89(122.90,124.88)	120.01(119.07,120.95)	117.08(116.16,118.00)

^a^ Mean (95% confidence interval), g/L. ^b^ Considering that there are more than 50 ethnic groups in China, other groups are not listed individually.

**Table 5:** The prevalence of anemia (%) in subgroups according to trimester.

Characteristics	Anemia in the 1st Trimester				Anemia in the 2nd Trimester				Anemia in the 3rd Trimester			
	Mild	Moderate	Severe	Total	Mild	Moderate	Severe	Total	Mild	Moderate	Severe	Total
Year *n* (%)												
2015	408 (2.88)	123 (0.87)	4 (0.03)	535 (3.77)	814 (8.12)	225 (2.24)	2 (0.02)	1041 (10.39)	2017 (17.20)	472 (4.02)	10 (0.09)	2499 (21.31)
2016	745 (3.92)	271 (1.43)	2 (0.01)	1018 (5.36)	1241 (7.66)	478 (2.95)	7 (0.04)	1726 (10.65)	3064 (17.99)	984 (5.78)	5 (0.03)	4053 (23.80)
2017	1225 (5.58)	370 (1.69)	29 (0.13)	1624 (7.40)	2012 (12.19)	542 (3.28)	41 (0.25)	2595 (15.72)	2453 (16.89)	1005 (6.92)	22 (0.15)	3480 (23.96)
2018	406 (4.20)	339 (3.51)	4 (0.04)	749 (7.75)	1469 (7.45)	677 (3.43)	1 (0.01)	2147 (10.89)	1443 (15.93)	360 (3.98)	4 (0.04)	1807 (19.95)
Area *n* (%)												
East	876 (2.97)	666 (2.26)	25 (0.08)	1567 (5.32)	3373 (7.68)	1312 (2.99)	41 (0.09)	4726 (10.76)	1230 (42.80)	10 (23.80)	10 (0.03)	6857 (21.58)
Central	750 (3.98)	172 (0.91)	4 (0.02)	926 (4.92)	1175 (12.73)	323 (3.50)	1 (0.01)	1499 (16.25)	476 (16.60)	2 (4.80)	2 (0.04)	1263 (24.08)
West	1196 (6.89)	381 (2.19)	13 (0.07)	1590 (9.15)	1293 (8.05)	446 (2.78)	10 (0.06)	1749 (10.90)	1166 (40.60)	30 (71.40)	30 (0.16)	4175 (21.94)
Hukou *n* (%)												
Urban residents	2148 (4.38)	734 (1.50)	20 (0.04)	2902 (5.92)	4335 (9.77)	1144 (2.58)	19 (0.04)	5498 (12.39)	7066 (16.77)	1939 (4.60)	14 (0.03)	9019 (21.41)
Migrants	212 (2.89)	264 (3.60)	4 (0.05)	480 (6.55)	467 (3.13)	565 (3.79)	6 (0.04)	1038 (6.96)	764 (15.73)	249 (5.13)	3 (0.06)	1016 (20.91)
Rural residents	462 (4.96)	221 (2.37)	18 (0.19)	701 (7.53)	1039 (10.50)	372 (3.76)	27 (0.27)	1438 (14.53)	1551 (17.14)	684 (7.56)	25 (0.28)	2260 (24.98)
Age group *n* (%)												
15–19	19 (4.50)	10 (2.37)	0 (0.00)	29 (6.87)	45 (5.80)	34 (4.38)	3 (0.39)	82 (10.57)	74 (16.23)	38 (8.33)	0 (0.00)	112 (24.56)
20–24	421 (4.69)	182 (2.03)	3 (0.03)	606 (6.76)	939 (9.00)	399 (3.83)	4 (0.04)	1342 (12.87)	1389 (18.02)	488 (6.33)	8 (0.10)	1885 (24.45)
25–29	1262 (4.00)	510 (1.62)	22 (0.07)	1794 (5.69)	2678 (8.40)	867 (2.72)	19 (0.06)	3564 (11.17)	4213 (16.27)	1293 (4.99)	16 (0.06)	5522 (21.33)
30–34	761 (4.26)	363 (2.03)	10 (0.06)	1134 (6.36)	1541 (8.45)	540 (2.96)	17 (0.09)	2098 (11.50)	2653 (16.98)	751 (4.81)	11 (0.07)	3415 (21.85)
35–39	297 (4.98)	127 (2.13)	4 (0.07)	428 (7.18)	536 (8.04)	206 (3.09)	8 (0.12)	750 (11.24)	928 (16.92)	259 (4.72)	6 (0.11)	1193 (21.75)
40–49	62 (6.53)	27 (2.85)	3 (0.32)	92 (9.69)	102 (8.70)	35 (2.99)	1 (0.09)	138 (11.77)	124 (14.29)	43 (4.95)	1 (0.12)	168 (19.35)
Ethnics *n* (%)												
Han	2740 (4.27)	1186 (1.85)	42 (0.07)	3968 (6.18)	5748 (8.48)	2044 (3.02)	51 (0.08)	7843 (11.57)	9199 (16.79)	2806 (5.12)	41 (0.07)	12,046 (21.98)
Other	82 (5.58)	33 (2.24)	0 (0.00)	115 (7.82)	93 (6.53)	37 (2.60)	1 (0.07)	131 (9.19)	182 (14.67)	66 (5.32)	1 (0.08)	249 (20.06)
Education *n* (%)												
High school	660 (3.90)	373 (2.21)	11 (0.07)	1044 (6.18)	1430 (9.06)	597 (3.78)	21 (0.13)	2048 (12.97)	2205 (17.75)	772 (6.21)	15 (0.12)	2992 (24.08)
College	925 (4.26)	349 (1.61)	18 (0.08)	1292 (5.96)	2090 (9.60)	554 (2.54)	23 (0.11)	2667 (12.25)	3183 (16.17)	757 (3.85)	10 (0.05)	3950 (20.07)
Master	167 (4.07)	47 (1.15)	2 (0.05)	216 (5.27)	227 (7.18)	46 (1.45)	3 (0.09)	276 (8.73)	633 (18.48)	115 (3.36)	1 (0.03)	749 (21.87)
Other	1070 (4.65)	450 (1.96)	11 (0.05)	1531 (6.66)	2094 (7.36)	884 (3.11)	5 (0.02)	2983 (10.48)	3360 (16.38)	1228 (5.99)	16 (0.08)	4604 (22.45)
Primigravida *n* (%)												
Yes	1291 (3.83)	671 (1.99)	20 (0.06)	1982 (5.87)	2934 (7.24)	1210 (2.99)	21 (0.05)	4165 (10.28)	4512 (15.64)	1237 (4.29)	12 (0.04)	5761 (19.97)
No	1531 (4.79)	548 (1.72)	22 (0.07)	2101 (6.58)	2907 (10.14)	871 (3.04)	31 (0.11)	3809 (13.28)	4869 (17.90)	1635 (6.01)	30 (0.11)	6534 (24.02)
Pre-pregnancy BMI *n* (%)												
18.5~23.99	1945 (4.29)	868 (1.91)	35 (0.08)	2848 (6.28)	4045 (8.46)	1420 (2.97)	33 (0.07)	5498 (11.49)	6388 (16.70)	1949 (5.10)	23 (0.06)	8360 (21.86)
<18.5	527 (5.58)	217 (2.30)	1 (0.01)	745 (7.89)	922 (9.11)	362 (3.58)	6 (0.06)	1290 (12.75)	1284 (17.39)	438 (5.93)	4 (0.05)	1726 (23.37)
24~27.99	252 (3.01)	106 (1.27)	5 (0.06)	363 (4.34)	604 (7.45)	209 (2.58)	10 (0.12)	823 (10.15)	1295 (16.43)	365 (4.63)	13 (0.16)	1673 (21.22)
>=28	66 (3.19)	18 (0.87)	0 (0.00)	84 (4.06)	195 (7.78)	64 (2.55)	3 (0.12)	262 (10.45)	306 (15.47)	95 (4.80)	2 (0.10)	403 (20.37)
Unknown	32 (6.82)	10 (2.13)	1 (0.21)	43 (9.17)	75 (12.20)	26 (4.23)	0 (0.00)	101 (16.42)	108 (19.74)	25 (4.57)	0 (0.00)	133 (24.31)

**Table 6:** Associations between basic characteristics and hemoglobin concentrations (g/L) in pregnant women according to trimester.

Characteristics	Trimesters OR (95%Confidential Interval)		
	1st Trimester	2nd Trimester	3rd Trimester
Year (Ref. = 2015)			
2016	−1.714(−1.962, −1.465) ^d^	0.600(0.280, 0.920) ^d^	−0.608(−0.907, −0.308) ^d^
2017	−0.833(−1.117, −0.549) ^d^	−0.282(−0.609, 0.045)	1.314(0.987, 1.641) ^d^
2018	−1.059(−1.387, −0.730) ^d^	1.270(0.947, 1.593) ^d^	3.628(3.282, 3.974) ^d^
2019	−4.611(−5.367, −3.854) ^d^	3.499(3.068, 3.930) ^d^	7.179(6.671, 7.688) ^d^
2020	−12.147(−17.539, −6.755) ^d^	7.491(6.606, 8.377) ^d^	12.556(11.634, 13.477) ^d^
Area (Ref. = Eastern)			
Central	−2.146(−2.392, −1.899) ^d^	−2.281(−2.583, −1.979) ^d^	0.046(−0.328, 0.420)
Western	−2.664(−2.926, −2.402) ^d^	1.699(1.439, 1.958) ^d^	0.094(−0.159, 0.346)
Hukou (Ref. = Urban residents)			
Migrants	−0.245(−0.542, −0.053) ^d^	3.006(2.707, 3.305) ^d^	0.003(−0.373, 0.379)
Rural residents	−0.562(−0.818, −0.307)	−1.534(−1.826, −1.242) ^d^	−1.569(−1.867, −1.270) ^d^
Education (Ref. = High school)			
College	0.634(0.404, 0.863) ^d^	1.338(1.062, 1.614) ^d^	1.157(0.869, 1.446) ^d^
Master	−0.0176(−0.406, 0.372)	2.641(2.138, 3.144) ^d^	−0.507(−0.994, −0.019) ^c^
Other	0.164(0.066, 0.393)	0.262(0.010, 0.513) ^c^	−0.164(−0.446, 0.118)
Age	−0.080(−0.102, −0.059) ^d^	0.078(0.056, 0.101) ^d^	0.115(0.090, 0.140) ^d^
Ethnics ^a^ (Ref. = Han)			
Other	0.105(−0.466, 0.676)	0.620(−0.049, 1.289)	0.401(−0.293, 1.095)
Gravidity (Ref. = Multipara)			
Primigravid	−0.305(−0.489, −0.122) ^d^	−2.143(−2.357, −1.929) ^d^	−2.427(−2.649, −2.205) ^d^
Pre-pregnancy BMI ^b^ (Ref. = 18.5~23.99)			
<18.5	−1.255(−1.501, −1.009) ^d^	−0.862(−1.136, −0.588) ^d^	−0.791(−1.100, −0.483) ^d^
24~28	2.382(2.123, 2.641) ^d^	1.100(0.797, 1.402) d	0.742(0.442, 1.042) ^d^
≥28	3.822(3.335, 4.309) ^d^	1.091(0.576, 1.605) ^d^	0.477(−0.080, 1.034)
Unknown	−1.560(−2.560, −0.560) ^c^	−2.275(−3.287, −1.264) ^d^	−2.014(−3.053, −0.975) ^d^

^a^ Considering that there are more than 50 ethnic groups in China, other groups are not listed individually. ^b^ BMI Body Mass Index. ^c^ *p* < 0.05, from multiple linear regression model. ^d^ *p* < 0.001, from multiple linear regression model.

**Table 7:** Associations between basic characteristics and the anemia prevalence in pregnant women according to trimesters.

Characteristics	Trimesters (95%Confidential Interval)		
	1st Trimester	2nd Trimester	3rd Trimester
Geographical area (Ref.: Eastern China)			
Central China	0.939(0.859, 1.026)	1.347(1.261, 1.438) ^d^	1.075(1.002, 1.154) ^c^
Western China	1.788(1.652, 1.935) ^d^	0.811(0.763, 0.863) ^d^	0.938(0.895, 0.983) ^d^
Hukou (Ref.: Urban residents)			
Migrants	1.213(1.089, 1.351) ^d^	0.537(0.496, 0.581) ^d^	0.875(0.827, 0.926)
Rural residents	1.274(1.164, 1.395) ^d^	1.192(1.115, 1.274) ^d^	0.826(0.758, 0.901) ^d^
Ethics ^a^ (Ref.: Han)			
Other	0.908(0.746, 1.104)	0.881(0.734, 1.057)	1.081(0.939, 1.244)
Education (Ref.: High school)			
College	0.890(0.816, 0.971) ^c^	0.877(0.822, 0.937) ^d^	1.093(1.036, 1.154) ^d^
Master	0.871(0.746, 1.018)	0.600(0.524, 0.687) ^d^	0.935(0.887, 0.986)
Other	0.953(0.876, 1.036)	0.845(0.795, 0.898) ^d^	1.058(0.964, 1.161) ^c^
Gravidity (Ref.: Multipara)			
Primigravid	0.988(0.922, 1.059)	0.847(0.804, 0.892) ^d^	0.772(0.740, 0.806) ^d^
Age	1.027(1.019, 1.035) ^d^	0.994(0.988, 1.000) ^c^	0.987(0.982, 0.992) ^d^
Pre-pregnancy BMI ^b^ group (Ref: Normal weight(18.5 ≤ BMI < 24))			
Underweight(BMI < 18.5)	1.246(1.144, 1.356) ^d^	1.198(1.122, 1.280) ^d^	0.910(0.747, 1.109) ^c^
Overweight (24 ≤ BMI < 28)	0.676(0.604, 0.757) ^d^	0.779(0.720, 0.842) ^d^	1.000(0.816, 1.226)
Obesity (BMI ≥ 28)	0.656(0.525, 0.820) ^d^	0.761(0.667, 0.869) ^d^	0.873(0.712, 1.070) ^c^
Unknown	1.443(1.050, 1.982) ^c^	1.330(1.071, 1.652) ^c^	0.800(0.639, 1.002)

^a^ Because there are more than 50 ethnic groups in China, other groups are not listed individually. ^b^ BMI Body Mass Index. ^c^ *p* < 0.05, from the Logistic regression model. ^d^ *p* < 0.001, from the Logistic regression model.

## Data Availability

The data presented in this study are not publicly available due to the restrictions of the local ethnics committee and institutional data security and privacy policies. The data are accessible from the corresponding author (Yuan Wei) on reasonable request and after obtaining institutional and ethics committee’s approval.
